# Machine learning identified an Alzheimer’s disease-related FDG-PET pattern which is also expressed in Lewy body dementia and Parkinson’s disease dementia

**DOI:** 10.1038/s41598-018-31653-6

**Published:** 2018-09-05

**Authors:** Audrey Katako, Paul Shelton, Andrew L. Goertzen, Daniel Levin, Bohdan Bybel, Maram Aljuaid, Hyun Jin Yoon, Do Young Kang, Seok Min Kim, Chong Sik Lee, Ji Hyun Ko

**Affiliations:** 1 Department of Human Anatomy and Cell Science, Max Rady College of Medicine, Rady Faculty of Health Sciences, University of Manitoba, Winnipeg, Manitoba Canada; 2 Neuroscience Research Program, Kleysen Institute for Advanced Medicine, Health Sciences Centre, Winnipeg, Manitoba Canada; 3 Section of Neurology, Max Rady College of Medicine, Rady Faculty of Health Sciences, University of Manitoba, Winnipeg, Manitoba Canada; 4 Section of Nuclear Medicine, Department of Radiology, Max Rady College of Medicine, Rady Faculty of Health Sciences, University of Manitoba, Winnipeg, Manitoba Canada; 5 Department of Nuclear Medicine, College of Medicine, Dong-A University, Busan, South Korea; 6 Institute of Parkinson’s Clinical Research, Ulsan University College of Medicine, Seoul, South Korea; 7 Department of Neurology, Asan Medical Center, Seoul, South Korea

## Abstract

Utilizing the publicly available neuroimaging database enabled by Alzheimer’s disease Neuroimaging Initiative (ADNI; http://adni.loni.usc.edu/), we have compared the performance of automated classification algorithms that differentiate AD vs. normal subjects using Positron Emission Tomography (PET) with fluorodeoxyglucose (FDG). General linear model, scaled subprofile modeling and support vector machines were examined. Among the tested classification methods, support vector machine with Iterative Single Data Algorithm produced the best performance, i.e., sensitivity (0.84) × specificity (0.95), by 10-fold cross-validation. We have applied the same classification algorithm to four different datasets from ADNI, Health Science Centre (Winnipeg, Canada), Dong-A University Hospital (Busan, S. Korea) and Asan Medical Centre (Seoul, S. Korea). Our data analyses confirmed that the support vector machine with Iterative Single Data Algorithm showed the best performance in prediction of future development of AD from the prodromal stage (mild cognitive impairment), and that it was also sensitive to other types of dementia such as Parkinson’s Disease Dementia and Dementia with Lewy Bodies, and that perfusion imaging using single photon emission computed tomography may achieve a similar accuracy to that of FDG-PET.

## Introduction

Alzheimer’s disease (AD) is the most prevalent form of age-related dementia. Currently, and based on clinical and methodological consensus, fluorodeoxyglucose (FDG) positron emission tomography (PET) is the most widely used brain imaging modality that complements clinical diagnosis of AD^1^. AD patients exhibit declines in FDG uptake within the parietotemporal, frontal and posterior cingulate cortices when compared to normal age-matched controls. There exists, however, significant individual variation in patient PET signal expression which limits the use of a definitive threshold for FDG-PET in AD diagnosis. Therefore, clinical reading of FDG-PET images is usually based on subjective impressions of the relative hypo-metabolism in key anatomical brain regions. Consequently, no quantifiable biomarker-related numeric value is produced and, as a result, it is often concluded that reports are equivocal. In addition, the sensitivity of subjective readings at an early disease stage remains challenging, while relatively high accuracy can be achieved for more advanced stages^1^.

Alternative diagnostic approaches include the use of a PET radiotracer that binds to AD pathology-specific proteins such as beta-amyloid (Aβ) and phosphorylated tau. Since their invention, Pittsburgh Compound-B (PiB)^2^ and other second-generation Aβ radioligands have been heavily researched within the field of molecular imaging, particularly over the past decade. Meta-analysis on longitudinal PiB studies estimates 94.7% sensitivity but 57.2% specificity^3^ which implies high false positive rates of Aβ-PET. This confounding factor is inevitable since it has been estimated that approximately 20–30% of cognitively normal subjects exhibit abnormally high levels of high Aβ protein^4^. For this reason, the purpose of the recent on-going IDEAS study (Imaging Dementia-Evidence For Amyloid Scanning; http://www.ideas-study.org), in which the benefit of Aβ-PET will be evaluated for Medicare coverage, had to be limited to assessing Aβ status rather than AD status. A number of groups are currently investigating tau-imaging agents with promising results in some instances, but more rigorous testing needs to be done in upcoming years for these radioligands to be proven to be of any notable clinical significance that can be translated into clinical utility in a healthcare setting^5^.

In a combined effort involving multiple research centers in North America, the AD Neuroimaging initiative (ADNI) has collected the largest available multi-modal longitudinal brain imaging database for patients with AD, MCI and normal control subjects (http://www.adni-info.org). Utilizing the ADNI database, a number of studies constructed quantitative biomarkers that may aid AD diagnosis^6–12^. One of the limitations of ADNI-based studies are the lack of other types of dementia. For example, it is unknown if the above-mentioned AD-related biomarkers generated from ADNI’s FDG-PET database are only unique to AD or it is commonly expressed in patients with dementia with Lewy body (DLB), the most frequently misdiagnosed diseases as AD.

In this study, we have constructed machine-based imaging biomarkers using ADNI’s FDG-PET dataset. We used general linear model (GLM), subprofile modeling (SSM)^13^, and support vector machine (SVM). GLM is the most fundamental method for constructing a prediction model that classifies data. SSM is a form of principal component analysis (PCA), and it has proven to be useful for differential diagnosis and prognosis in other neurodegenerative disorders^14–16^. SVM is one of the most widely used machine learning algorithms that are used to solve binary classification problems with a large dataset^17^. We have compared the performance of these machine-based classification methods between AD and age-matched normal subjects, evaluated if they predict AD conversion from the prodromal state, examined if they are sensitive to other types of dementia such as DLB and Parkinson’s disease dementia (PDD).

## Results

### Performance Comparisons of different classification machines

**Figure 1:**
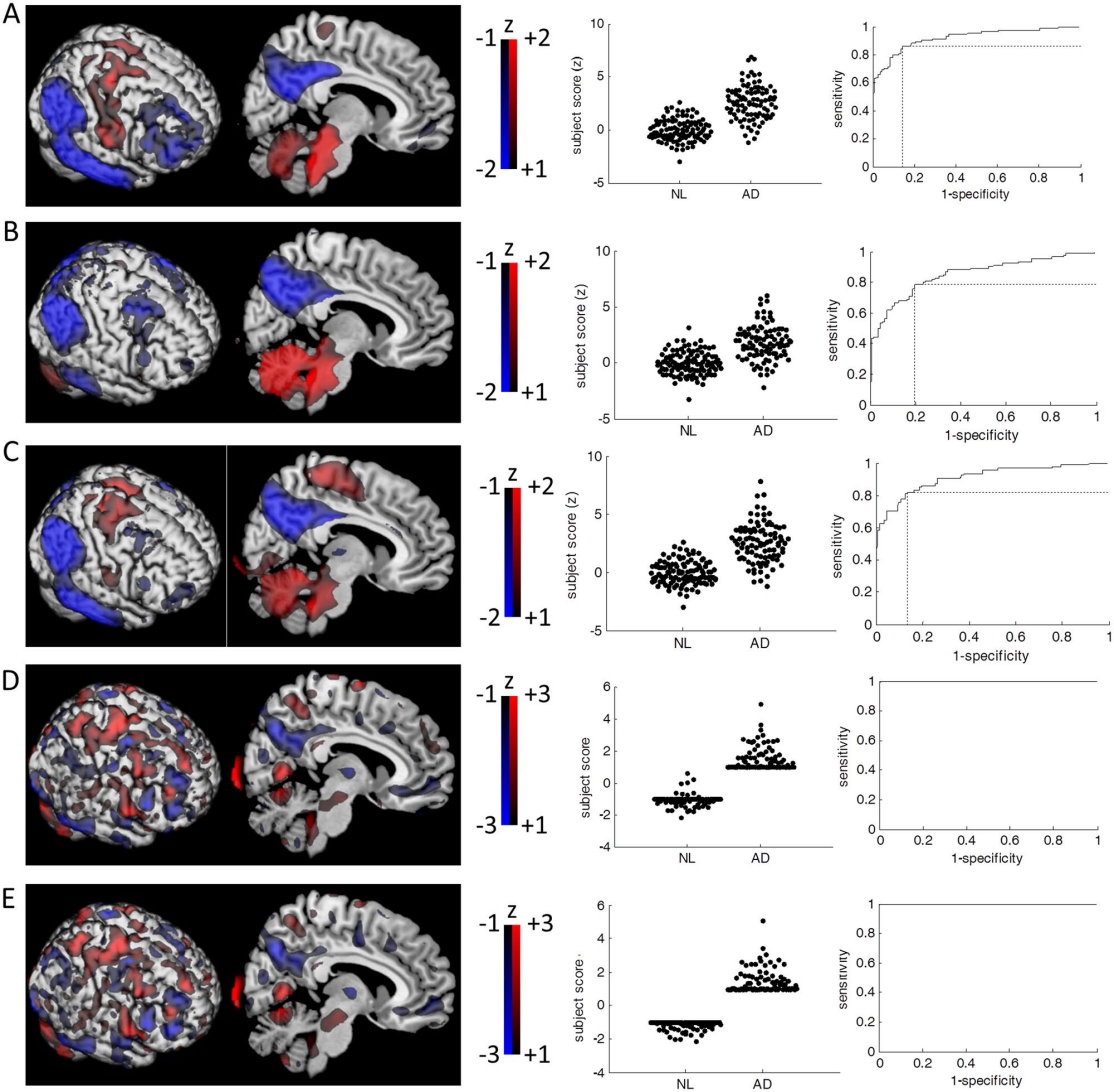
Visualization of classification machines. (**A**) Beta coefficient of voxel-wise linear regression (left). Subject scores are defined as the dot-product between the Beta coefficient map and the subject’s proportionally scaled FDG-PET images. The subject scores are z-scored to the mean and standard deviation of normal subjects (middle). Receiver-operating-characteristic (ROC) curve analysis is performed to estimate the area-under-the-curve (AUC; 0.922). The optimal subject score threshold for group classification is determined (z = 0.987) at the biggest sensitivity (0.862) × specificity (0.856). (**B**) In SSM/PCA1, the topography was similar to the GLM’s beta-coefficient map (left). The subject scores of the first PC (middle) produced the greatest separation of the groups (z-threshold = 0.771, AUC = 0.852, sensitivity = 0.787, specificity = 0.802; right). (**C**) The combined PCs of SSM/PCA2 was topographically similar to both GLM’s beta coefficient map and the first PC (left). The PCs were weighted by stepwise regression coefficients that significantly improved the model (p < 0.05). The resulting combined PC was 4.204 × PC1 + 0.865 × PC2 + 1.126 × PC3 – 1.825 × PC4 + 1.470 × PC5 + 1.099 × PC7 + 1.118 × PC10. The group separation by subject scores (middle) were relatively better than SSM/PCA1 of the first PC (z-threshold = 1.101, AUC = 0.911, sensitivity = 0.819, specificity = 0.865; right). (**D**) The hyperplane constructed by SVM-ISDA was topographically distinct from GLM or SSM/PCA (left). While complete group separation was achieved (AUC = 1; right), 3 of 111 NL subjects were labeled as AD (middle). It should be noted, in SVM’s case, that no score threshold is determined by ROC curve, but the sign of dot-product between hyperplane and subjects FDG-PET image (proportionally scaled) determines the labelling. (**E**) The hyperplane constructed by SVM-SMO (left) was topographically similar to the SVM-ISDA’s. Complete group separation was achieved (middle/right). For visualization purposes, all images are z-scored to the mean and standard deviation of all the voxel values of the whole-brain.

**Figure 2:**
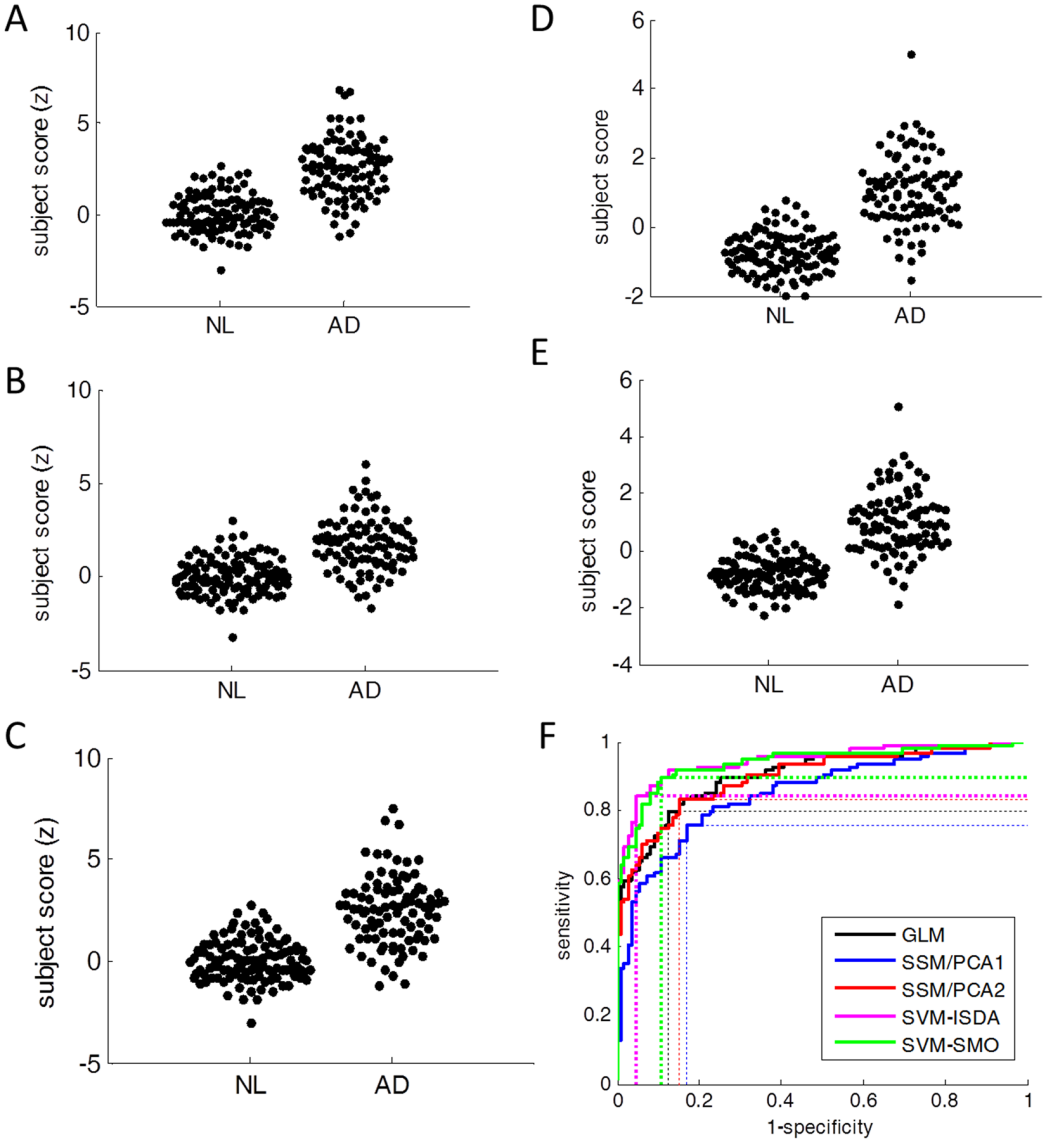
Subject scores determined by 10-fold cross-validation. (**A**) GLM, (**B**) SSM/PCA1, (**C**) SSM/PCA2, (**D**) SVM-ISDA and (**E**) SVM-SMO. (**F**) ROC analysis showed that AUC was the greatest in SVM-ISDA.

From the ADNI database, we identified 94 patients with AD and 111 age-matched control subjects who stayed cognitively healthy for >3 years after the FDG-PET scans (Table 1). We have investigated the classification performance of three different approaches; voxel-wise GLM (Fig. 1A), SSM/PCA and SVM. Two different approaches of SSM/PCA were utilized^13^; 1) selecting the single principal component (PC) that best discriminates the groups (SSM/PCA1); and 2) linear combination of PCs that significantly improved the model by stepwise regression (SSM/PCA2). In SSM/PCA1, the first PC (PC1) discriminated the two groups most significantly (Fig. 1B). In SSM/PCA2, 7 PCs were linearly combined (PC1-5, PC7 and PC10) with the biggest coefficient being PC1 (Fig. 1C). In SVM analysis, two different solvers were used: (1) Iterative Single Data Algorithm (ISDA^18^; Fig. 1D) and (2) sequential minimal optimization (SMO^19^; Fig. 1E).

**Table 1:** Demographic data for patients acquired from the ADNI (Alzheimer’s Disease Neuroimaging Initiative) database.

	NL	AD	Stable MCI	Prodromal AD
Number of patients	111	94	186	55
Age	75.3 (6.4), 63–94	75.5 (8.3), 56–90	71.2 (7.7), 55–90	*75.1 (6.5), 60–87
MMSE	29.0 (1.1), 26–30	*24.2 (1.8), 20–26	28.2 (1.6), 24–30	*26.9 (1.9), 24–30
Sex (M:F)	91:107	59:35	100:86	33:22
Follow-Up Duration	57.2 (20.9), 36–121	*10.4 (7.1), 0–27	53.7 (18.2), 36–112	*40.7 (23.7), 0–107

*p < 0.05, student t-test between NL vs. AD and stable MCI vs. prodromal AD. Sex ratio is compared by chi-square test.

Qualitatively, the produced patterns consistently show a well-characterized AD-like distribution of hypometabolism^20^ present within the precuneus, medial frontal lobes, the temporal lobes, and the cingulum (posterior and anterior). Relative hypermetabolism was observed in the somatosensory-motor areas, basal ganglia, thalamus and cerebellum. Our findings echo those observed in the previously identified AD-related metabolic patterns^21^. Quantitatively, the whole-brain metabolic patterns from GLM and SSM/PCA were topographically similar to each other (p < 0.01, corrected for autocorrelation^22^ and multiple comparisons), but the similarities were not significant when compared to the SVM-produced patterns (p > 0.1 corrected for autocorrelation^22^ and multiple comparisons; Table 2).

**Table 2:** Topographical similarities across different whole-brain metabolic patterns that distinguishes AD from normal subjects.

	GLM	SSM/PCA1	SSM/PCA2	SVM-ISDA	SVM-SMO
GLM	1	*0.829	*0.924	0.392	0.381
SSM/PCA1	*0.829	1	*0.896	0.213	0.201
SSM/PCA2	*0.924	*0.896	1	0.296	0.283
SVM-ISDA	0.392	0.213	0.296	1	*0.957
SVM-SMO	0.381	0.201	0.283	*0.957	1

*Significant similarities determined by topographical correlation method corrected for auto-correlation^22^ and Bonferroni multiple comparisons (p < 0.05).

As expected, all five approaches reliably discriminated patients and controls (sensitivity > 0.78, specificity > 0.80). When compared with 10-fold cross-validation (Fig. 2), the subject scores of the first three methods were almost identical to the original scores shown in Fig. 1 in AD (GLM: r = 0.998, r < 0.001; SSM/PCA1: r = 0.986, p < 0.001; SSM/PCA2: r = 0.990, p < 0.001) as well as in NL (GLM: r = 0.998, r < 0.001; SSM/PCA1: r = 0.992, p < 0.001; SSM/PCA2: r = 0.994, p < 0.001). While strongly correlated, lesser agreement was observed between original subjects scores and the ones from 10-fold cross-validation in SVM methods in both AD (SVM-ISDA: r = 0.860, p < 0.001; SVM-SMO: r = 0.875, p < 0.001) and in NL (SVM-ISDA: r = 0.648, p < 0.001; SVM-SMO: r = 0.739, p < 0.001), suggesting some overfitting in SVM. Nevertheless, the area-under-the-curve (AUC) for receiver-operating-characteristic (ROC) plot was the greatest by SVM-ISDA by 10-fold cross-validation (Fig. 2F; Table 3). Utilizing the structural MRI in the spatial normalization during the pre-processing did not improve the classification performance^23^ (Supplementary Table S1).

**Table 3:** Performance of AD vs. Normal classification methods using ADNI data by 10-fold cross-validation.

	GLM	SSM/PCA1	SSM/PCA2	SVM-ISDA	SVM-SMO
AUC	0.902	0.849	0.897	*0.945	0.939
Sensitivity	0.798	0.755	0.830	0.840	*0.894
Specificity	0.874	0.829	0.847	*0.955	0.892

*The best performance.

### Relationship with other risk factors

**Figure 3:**
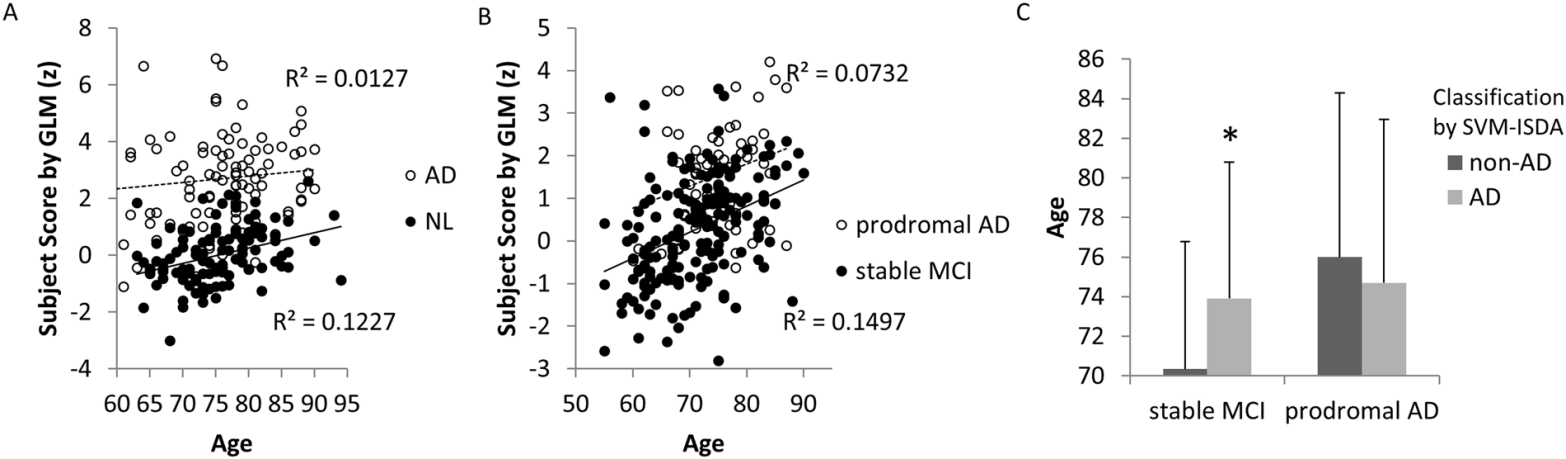
Effects of age. (**A**) In NL, age was significantly correlated with subject scores determined by GLM method (r = 0.350, p < 0.001). In AD, no correlation was observed (r = 0.113, p = 0.279). Similar level of correlations were also observed only in NL when SSM/PCA1 (r = 0.370, p < 0.001) and SSM/PCA2 (r = 0.367, p < 0.001) were used. (**B**) Likewise, similar level of correlations between subject scores and age were observed only in stable MCI (GLM: r = 0.387, p < 0.001; SSM/PCA1: r = 0.329, p < 0.001; SSM/PCA2: r = 0.401, p < 0.001) but not in prodromal AD (r < 0.28). (**C**) In stable MCI, patients who were designated as “AD” (n = 45 out of 141) by SVM-ISDA were significantly older than patients who were designated as “non-AD” (^*^t(184) = 2.762, p = 0.006). Similar trend was observed when SVM-SMO was used (t(184) = 1.767, p = 0.079). This age difference associated with “AD”-designation was not observed in prodromal AD patients in both SVM methods (p > 0.48).

**Figure 4:**
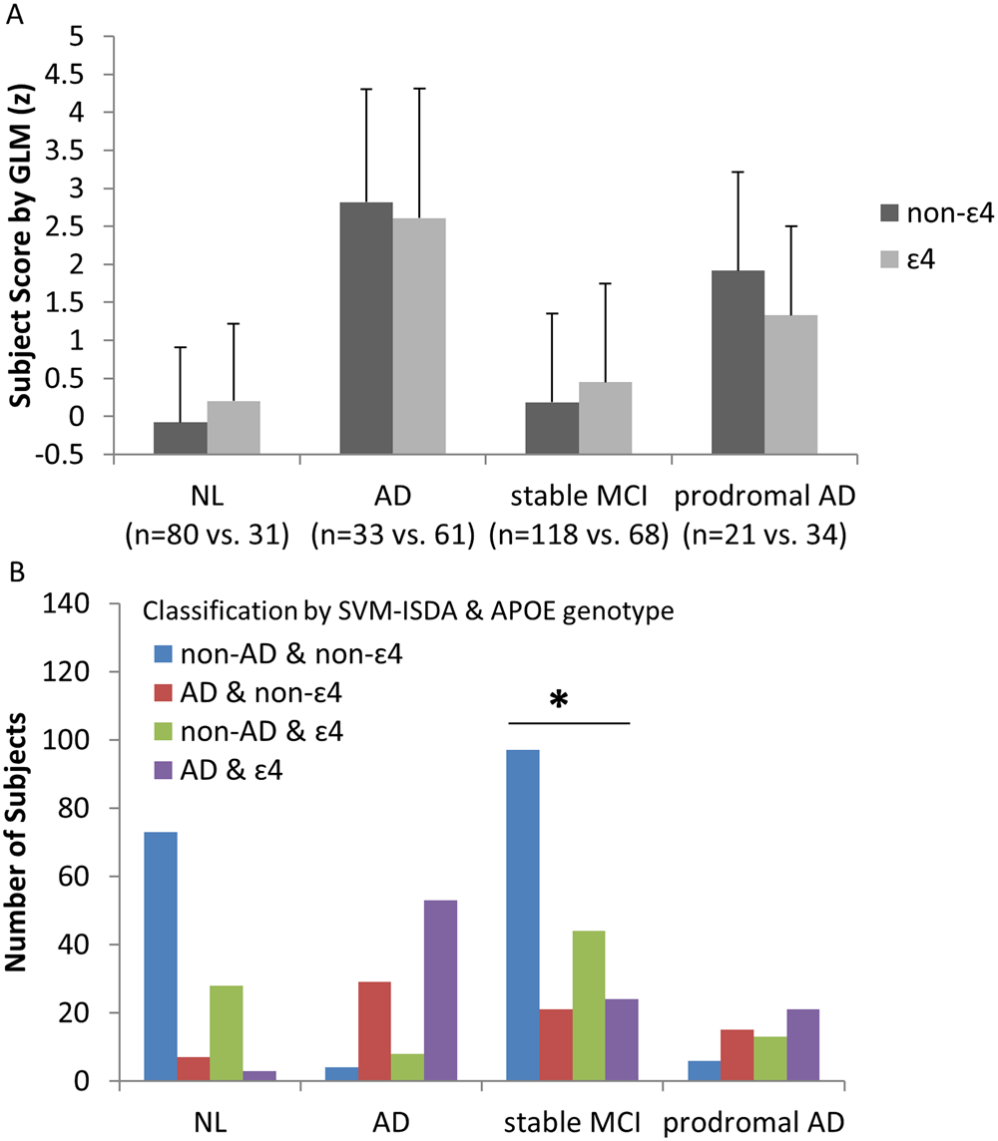
Effects of APOE mutation. (**A**) When GLM was used, no significant effects of APOE genotype in subject scores were observed in any groups (p > 0.08, student t-test), which was replicated when SSM/PCA was used (p > 0.06). (**B**) The proportion of subjects classified as “AD” vs. “non-AD” by SVM-ISDA was significantly different between APOE ɛ4 carriers vs. non-carriers only in stable MCI patients (χ^2^ = 7.202, p = 0.007, Pearson Chi-Square). In other words, if a patient with stable MCI has ɛ4, it is more likely that the patient will be classified as “AD”. From the 118 stable MCI non-ɛ4 carriers, 21 patients (17.8%) were classified as AD, in contrast to 24 of 68 stable MCI ɛ4 carriers (35.3%) were classified as AD. Similar results were produced when SVM-SMO was used in stable MCI patients (χ^2^ = 10.690, p = 0.001). In contrast, 8–10% normal control subjects received AD-designation regardless of APOE status. In AD, 86–88% individuals received AD-designation regardless of APOE status. In prodromal AD, 61–72% individuals received AD-designation regardless of APOE status.

In normal control subjects, age was significantly correlated with subject scores determined by GLM (r = 0.350, p < 0.001, Pearson’s correlation; Fig. 3A), SSM/PCA1 (r = 0.370, p < 0.001) and SSM/PCA2 (r = 0.367, p < 0.001). Since SVM outcome is dichotomous, student t-test was performed for age between subjects who were classified as AD vs. non-AD within normal control (SVM classified AD vs. NL in 100% accuracy in the training set thus the AD-designation determined as a testing set in 10-fold cross-validation was used). Age was not significantly associated with SVM’s designation of AD (p > 0.1, student t-test). The subject scores determined by GLM, SSM/PCA1 and SSM/PCA2 were not correlated with any of the other variables (MMSE, Aβ, tau and phosphorylated tau; p > 0.12, Pearson’s correlation) and they were not significantly different between sexes (p > 0.09, student t-test) or the presence of APOE ɛ4 genotype (p > 0.15, student t-test; Fig. 4A-left). These variables were not associated with SVM’s AD designation, either (p > 0.11, student t-test or chi-square; Fig. 4B-left).

The same analysis was repeated in AD, and no significant association was found between FDG-PET-based (subject scores and AD-designation) and other variables (age, MMSE, Aβ, tau and phosphorylated tau, sex, APOE genotype; p > 0.19). No significant effect of anti-AD medication status was observed in subject scores determined by GLM, SSM/PCA1 and SSM/PCA2 (p > 0.83, student t-test) or AD-designation by SVM-ISDA and SVM-SMO (p > 0.86, chi-square).

### Prediction of AD conversion at MCI stage

**Figure 5:**
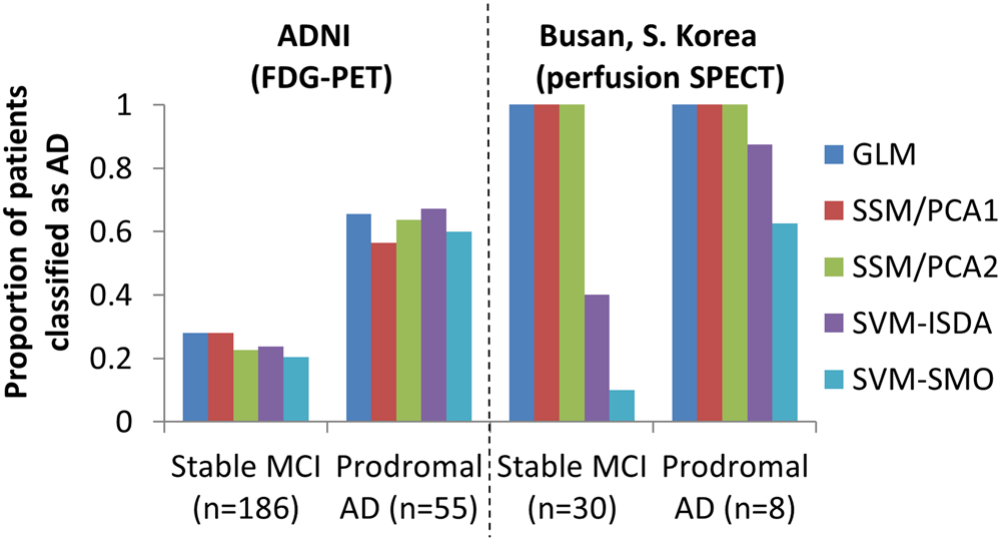
Prediction of future development of AD. Five different classification machines were applied to predict future development of AD from MCI patients who have been followed for >3 years. In the ADNI cohort, consistent group classification performance was achieved across 5 different approaches (left). When the perfusion SPECT data was used, GLM, SSM/PCA1 and SSM/PCA2 classified both prodromal AD and stable MCI patients as AD. Desirable performance (sensitivity × specificity > 0.5) was achieved in both SVM methods (right). It should be noted that the AD diagnosis was based on clinical examination, thus misdiagnosis cannot be ruled out, and may have contributed to the low sensitivity. It should be also noted that the limited follow-up period may have hindered the specificity of the method, i.e., if a longer follow-up was available, some of the stable MCI patients who have been classified as AD may have actually developed AD.

When the same machines were applied to predict future development of AD from 241 MCI patients (55 of whom developed AD and 186 of whom did not for the next 3 years; Table 1 - ADNI), a moderate sensitivity (0.563–0.673) and specificity (0.720–0.796) was achieved across all five different approaches (Fig. 5-left), which was comparable to prior studies^7,24^.

As in NL, age was correlated with subject scores (or AD-designation by SVM) in stable MCI patients (p < 0.001; Pearson’s correlation for GLM and SSM/PCA and student t-test for SVM; Fig. 3B,C), while Aβ, tau or phosphorylated tau concentrations were not associated with subject scores (p > 0.2, Pearson’s correlation for GLM and SSM/PCA) and AD-designation (p > 0.11, student t-test). APOE genotype was not associated with subject scores determined by GLM or SSM/PCA (p > 0.16, student t-test), but significantly more proportion of stable MCI patients had APOE ɛ4 allele when they were designates as AD than non-AD by both SVM methods (p < 0.007, chi-square; Fig. 4B).

In prodromal AD, age was very weakly correlated with GLM score (r = 0.271, p = 0.046; Fig. 3B) but not with SSM/PCA’s (p > 0.07). These subject scores were correlated with Aβ (r = −0.720, p = 0.019), tau (r = −0.595, p = 0.070) and phosphorylated tau (r = −0.749, p = 0.013) concentrations. Here, it should be noted that only 10 out of 55 prodromal AD patients had CSF measurements. As in AD, no significant difference was observed between the AD-designated patients and non-AD-designated patients in age, MMSE, Aβ, tau, phosphorylated tau (p > 0.075, student t-test), or APOE genotype (p > 0.3, chi-square), by either SVM methods.

Interestingly, when applied to the perfusion SPECT images from 38 patients with MCI (8 of whom later developed AD; 3-year follow-up; data from Dong-A University, Busan, S. Korea; Table 4), all patients (both stable MCI and prodromal AD) were classified as AD in GLM and SSM/PCA (Fig. 5-right). When SVM-ISDA was used, a higher level of sensitivity (0.875) was achieved but specificity was slightly poorer (0.600). When SVM-SMO was used, a similar level of sensitivity (0.625) and higher specificity (0.900) to the ADNI data was achieved. This result suggests a possibility that perfusion imaging may be as useful as FDG-PET for complementing AD diagnosis when analyzed by an advanced machine learning approach^25^.

**Table 4:** Demographic data for patients referred to the SPECT centre at the DongA University Hospital in Busan, S. Korea.

	Stable MCI	Prodromal AD
Number of patients	30	8
Age	68.0 (7.3), 55–80	71.8 (7.3), 58–80
MMSE	25.8 (2.7), 20–30	*22.5 (2), 20–25
Sex (M:F)	12:18	4:4

*p < 0.05, student t-test between stable MCI vs. prodromal AD. Sex ratio is compared by chi-square test.

### Clinical Applications

**Figure 6:**
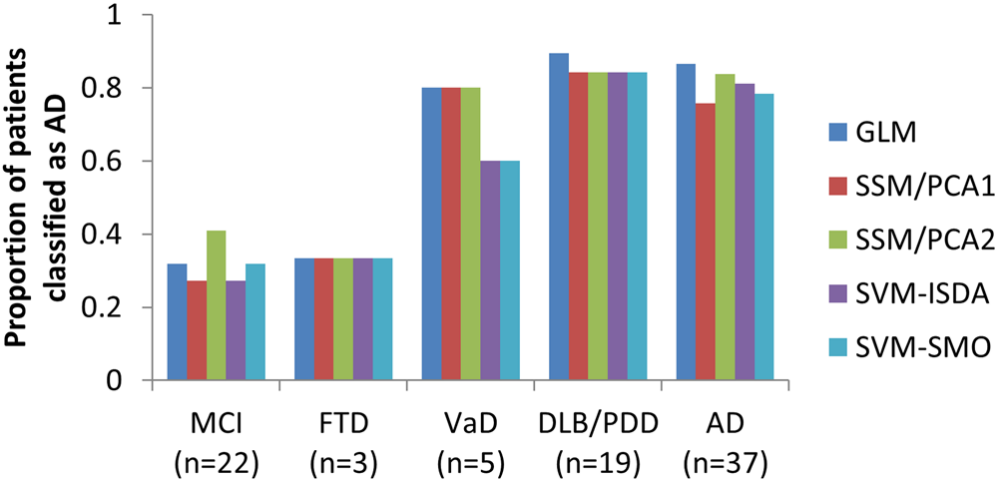
Patient classification performance in a real clinical setting. Four out of five approaches (GLM, SSM/PCA1, SVM-ISDA and SVM-SMO) achieved desirable performance (sensitivity × specificity >0.5) in classifying prodromal AD vs. stable MCI. Interestingly, >84% of DLB/PDD patients were also classified as “AD”, while low sample size limits definite conclusion on the cases of FTD and VaD.

In order to demonstrate the applicability of our proposed classification methods in a real clinical setting, a retrospective imaging study was conducted. In our picture archiving and communication system (PACS) at Health Science Centre (Winnipeg, Manitoba), we have identified 113 patients who referred to brain FDG-PET between 2010 and 2012 (Table 5). All patients were referred from our local memory clinic due to subjective memory complaints or MCI. Among these patients, 37 patients were subsequently diagnosed with AD, 19 patients with DLB or PDD, 4 patients with frontotemporal dementia (FTD; 1 patient excluded due to image quality issue), and 5 patients with vascular dementia (VaD). 21 patients received other diagnoses during the follow-up period. 18 patients did not develop any forms of dementia for >6 months, and 6 patients did not return for follow-up (thus excluded from our analysis). All five different approaches (GLM, SSM/PCA1, SSM/PCA2, SVM-ISDA and SVM-SMO) accurately classified the majority of patients who were later diagnosed with AD as AD (28–32 of 37; Fig. 6). The majority of patients who did not develop any form of dementia for >6 months were accurately classified as non-AD (13–16 of 22 stable MCI patients). Interestingly, most patients (16–17 of 19) who later diagnosed with DLB or PDD were also classified as AD, suggesting non-specificity of our method in different types of dementia, especially in alphasynucleinopathy-related dementia (PDD and DLB). The number of patients with FTD (n = 3) and VaD (n = 5) were very small, thus no formal conclusion can be drawn from this data. Nevertheless, more frequent “AD-designation” was observed in patients who were later diagnosed with VaD (3–4 of 5).

**Table 5:** Demographic data for patients referred to the PET Centre at the Health Sciences Centre in Winnipeg, Manitoba, Canada.

	MCI	AD	DLB/PDD	FTD	VaD
Number of patients	22	37	19	3	5
Age	69.8 (5.4), 61–78	68.9 (8.7), 50–84	68.1 (7.7), 56–81	71 (11.4), 63–84	73.0 (10.8), 58–86
MMSE	26.8 (1.9), 22–29	*24.4 (3.4), 16–29	25.1 (3.5), 14–30	26.3 (3.8), 22–29	25.0 (3.0), 22–30
Sex (M:F)	9:13	16:21	9:10	2:1	3:2
Follow-Up Duration	25.1 (19.3), 6–59	34.4 (22.6), 2–83	*37.4 (16.6), 8–66	19.7 (11.3), 7–29	36.4 (15.9), 11–52

*p < 0.05, student t-test between MCI and other disease group. Sex ratio is compared by chi-square test. AD patients and other disease groups are not significantly different.

### AD-related metabolic pattern in DLB and PDD

**Figure 7:**
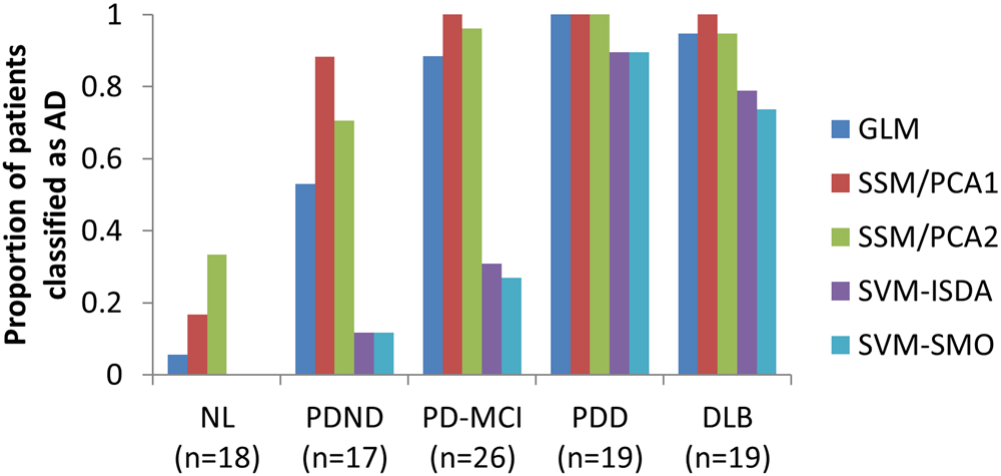
AD-like glucose metabolic pattern expression in alphasynucleinopathy. As can be expected from Fig. 3, the majority of PDD (17 of 19) and DLB (14–15 of 19) patients were classified as AD, when analyzed by the SVM method. However the majority of non-demented PD (PDND; 15 of 17)) or PD patients with MCI (PD-MCI; 18–19 of 26) were not classified as AD. None of the healthy control subjects were classified as AD when SVM was used. When other methods (GLM and SSM/PCA) were used, the rate of AD designation was significantly higher in PD-MCI, PDND and NL, potentially suggesting false positive identification.

In order to investigate the comorbid expression of an AD-like brain metabolic pattern in Parkinson’s disease (PD), we revisited the FDG-PET images from Asan Medical Centre (Seoul, S. Korea; Table 6)^26^. When SVM was used, none of the healthy control subjects (n = 18) were classified as AD, and only 2 of 17 non-demented PD (PDND) patients were classified as AD (Fig. 7). This rate was slightly increased in PD patients with MCI (7–8 of 26), while the majority of PDD (17 of 19) and DLB (14–15 of 19) patients were classified as AD (Fig. 4). The other methods (GLM, SSM/PCA1 and SSM/PCA2) were generally more sensitive but false positive identification from normal control subjects (1–6 of 18) was also more frequent (Fig. 7). Together with the above findings (Fig. 6), this result suggests that both PDD and DLB patients exhibit AD-like brain metabolic patterns while cognitively healthy PD patients do not (when examined by SVM).

**Table 6:** Demographic data for patients from Movement Disorder Clinic at Asan Medical Centre in Seoul, S. Korea.

	NL	PDND	PD-MCI	PDD	DLB
Number of patients	18	17	26	18	18
Age	59.7 (6.9), 46–73	^$^65.8 (11.1), 41–85	*71.3 (5.1), 62–80	*70.7 (5.1), 58–78	*73.7 (5.7), 66–86
MMSE		^$^28.4 (1.2), 27–30	^$^22.3 (2.7), 16–26	16.2 (6.6), 3–24	18.3 (4.6), 11–25
Sex (M:F)	3:15	*10:7	*16:10	8:10	8:10

*p < 0.05, student t-test between NL and other disease group. ^$^p < 0.05, student t-test between DLB and other disease group. Sex ratio is compared by chi-square test.

## Discussion

When FDG-PET was used, the best classification performance (sensitivity × specificity) was achieved by the SVM-ISDA in all three cases (AD vs. NL from ADNI by 10-fold cross-validation; stable MCI vs. prodromal AD from ADNI; stable MCI vs. prodromal AD from Winnipeg) among the five different classification methods (Table 7). Thus, we recommend the use of SVM-ISDA in FDG-PET readings to complement AD diagnosis.

**Table 7:** Overall performance (sensitivity × specificity) of classification machines.

Group Classification	Dataset	GLM	SSM/PCA1	SSM/PCA2	SVM-ISDA	SVM-SMO
^$^AD vs. NL	ADNI	0.675	0.626	0.703	*0.803	0.797
stable MCI vs. prodromal AD	ADNI	0.472	0.406	0.493	*0.514	0.477
	Winnipeg	*0.590	0.550	0.495	*0.590	0.534
	Busan (SPECT)	0.000	0.000	0.000	0.525	*0.563

*The best performance.

^$^Since this is the training set of the classification machines, the AD designation was based on 10-fold cross-validation.

Of note, the GLM and SSM/PCA produced topographically similar metabolic patterns. This is not unexpected when a large training set was used (Eidelberg, personal communication). The region weights, in both GLM and SSM/PCA, are indicators of overall metabolic topography that differentiates AD from NL. In other words, the subject scores are computed as a dot-product between patients’ normalized FDG-PET (or perfusion SPECT) images and the metabolic “template” images (Fig. 1A–C). Thus, it quantitates to what degree subjects’ images are “look-a-likes” of those template images. And, this gives one a rationale to use it as a quantifiable biomarker to correlate with disease progression, symptom severity or treatment responses^27^. Indeed, the subject scores determined by GLM and SSM/PCA were significantly correlated with age in normal subjects and stable MCI, consistent with old age being one of the most prominent risk factor for AD.

In SVM, higher weights are assigned to the hyperplanes (Fig. 1D,E) if the value of the voxel (feature) is useful for group separation. In other words, the “importance” of each feature (i.e., voxel) is weighted in the hyperplane. And, the subject score itself is computed in the same manner as in GLM and SSM/PCA, i.e., dot-product with the subjects’ normalized images and the hyperplane. The difference is that only the sign of the subject score is used for group classification. Thus, the absolute size of the subject scores does not convey the same level of information (e.g., age or disease status) as the GLM and SSM/PCA does, but it only bears information about the dichotomic class designation. In this regard, it is not surprising that SVM-produced subject scores were not associated with age while GLM and SSM/PCA-produced subjects scores were. This fundamental difference resulted in a spatially less smooth representation of AD-related metabolic pattern, and dissimilar topographical profile from GLM and SSM/PCA (Table 2). Nevertheless, qualitatively, all five approaches were able to pinpoint relative hypometabolism in key brain regions commonly effected by AD: the occipito-parietal, frontal and temporal lobes and the cingulum^28^.

The robust performance of SVM-ISDA classification of prodromal AD vs. stable MCI patients from three different datasets (Figs 5 and 6), including the data archived from our community-based memory clinic demonstrates its immediate translational value. Interestingly, most patients with other forms of dementia (i.e., clinically determined during the follow-up period) also show positive AD designation. The DLB/PDD patients, especially, showed even higher proportion of positive results than AD patients. This was not surprising since DLB/PDD and AD often present with very similar clinical symptoms and neuropathology. Amyloid deposits and tau protein accumulation are commonly observed in patients with DLB, and it has been well established that these AD-related pathologies are also linked to the cognitive deficits that occur in DLB patients^29^. The similar neurodegenerative etiology of the two is also often reflected in FDG-PET scans^30^. The hypometabolic pattern observed in DLB is highly congruent with and can be easily superimposed onto that observed in AD patients with the only region exhibiting variation between the two being the occipital lobe^31^. The clinical overlap makes the two diseases fairly difficult to distinguish and accurate differential diagnosis between AD and DLB remains a popular arena of neurodegenerative disease research, and it is warranted that an additional step be taken for differential diagnosis, e.g., Parkinson’s disease-related metabolic pattern expression may be examined in these patients^26^. Other PET/SPECT radiotracers that target the dopaminergic^32^ system or tau^33^ have shown promising results for differentiating DLB from AD.

More interestingly, PDND and PD-MCI patients were rarely classified as “AD” by SVM-ISDA (Fig. 4). Taken together with the high rate of “AD-classification” of prodromal PDD/DLB patients (from HSC data), this result demonstrates a very important translational potential not only for predicting AD but also predicting PDD from non-demented PD patients. According to the dual syndrome hypothesis, PDD/DLB is more involved with posterior and temporal brain regions associated with visuospatial and attention deficits while non-progressing PD-MCI is more involved with fronto-subcortical circuits associated with executive function^34–37^. It is of interest that those visuospatial and attention deficits respond relatively well to the cholinergic treatment commonly used in AD^36^, prompting common pathology between AD and PDD/DLB. Nevertheless, there are autopsy-confirmed PDD patients without distinct AD-like pathology^38^. We have recently reported a brain metabolic pattern that was associated with cognitive decline in PDD/DLB, which was characterized by a hypometabolic caudate nucleus^39^. PDD/DLB patients also show further decreased caudate FP-CIT uptake (dopamine transporter availability) compared to the PDND patients^26^. Interactions between those two domains, AD-like vs. PDD/DLB-specific (e.g., involving the caudate nucleus) remain to be elucidated.

It is yet unknown why GLM and SSM/PCA designated more PDND and PD-MCI patients as AD (52–100%) while SVM didn’t (11–31%). One potential explanation is that the GLM and SSM/PCA-derived AD-related glucose metabolic patterns share some topographical property of “normal” aging-related metabolic pattern^40,41^ potentially due to the parallel processes between aging and AD (and other neurodegenerative disorders such as PD)^42^. Thus, it is possible that severer AD patients also showed higher aging-related changes, and their scans would also have contributed more to pattern topography. Of note, age was weakly but significantly correlated with GLM and SSM/PCA-derived pattern expression in normal control. On the other hand, SVM does not take the severity of the disease into consideration but instead focuses on the boundary between the two groups. The individuals who were used as support vectors are the ones who are adjacent to the marginal space. In other words, the hyperplane is formed based on the AD patients whose scans are better normal individual “look-a-likes”, as well as the normal individuals whose scans are better AD patient “look-a-likes”. Perhaps, the normal aging-related metabolic changes are similarly expressed in these two subpopulations, thus cancelling out when the hyperplane was defined. It must be noted that the SVM-ISDA is only designed for group separation and is therefore not designed to convey any information about disease status. For the purposes of monitoring the disease progression and/or treatment responses, GLM and SSM/PCA may be more suitable choices. Nevertheless, it is of interests that only the SVM’s AD designation was associated with APOE genotype in stable MCI, which is in agreement with the APOE ɛ4 status being one of the most significant risk factor for AD^43^, e.g., only 31 of 111 normal controls (28%) were ɛ4 carrier in contrast to 46 of 74 AD patients (61%).

When perfusion SPECT (from Dong-A University Hospital) was analyzed with SVM-ISDA, more prodromal AD patients were designated as AD (87%) than when FDG-PET (from ADNI) was analyzed (67%). This is in line with the recent observation that vascular changes precede metabolic changes^44^. However, it should be noted that there were also more false positives in perfusion SPECT, i.e., 40% of stable MCI were designated as AD in perfusion SPECT while only 24% were designated as AD in FDG-PET. Without longer follow-up duration and larger sample size, it is difficult to draw the conclusion that one method (perfusion SPECT) was superior to others (FDG-PET). It should be also noted that when SVM-SMO was used for analysis, perfusion SPECT’s sensitivity and specificity were similar to that of FDG-PET from the ADNI’s MCI patient database.

## Materials and Methods

Data used in the preparation of this article were obtained from the Alzheimer’s Disease Neuroimaging Initiative (ADNI) database (adni.loni.usc.edu). ADNI was launched in 2003 as a public-private partnership, led by Principal Investigator Michael W. Weiner, MD.

### Subjects

A total of 763 subjects from the ADNI database who had available PET and MRI scans were included in this study (Supplementary Fig. S1). ADNI subjects came from one of three groups: AD, normal controls (NL), and MCI. MCI patients were further divided into stable MCI (who did not develop AD during the 3 years of follow-up period) and prodromal AD (who developed AD within 3 years). MCI patients who were not followed for more than 3 years were excluded. Demographic details regarding the ADNI cohort are given in Table 1.

To evaluate the utility of the proposed methods’ early diagnostic capacity using different imaging modalities, data from a total of 38 patients with MCI who underwent perfusion SPECT (TC-99m HMPAO) at Dong-A University Hospital in Busan, South Korea, then were subsequently followed for clinical diagnosis (>3 years) were included (Table 4). Patients were followed periodically over 1–2 years to monitor progression to dementia status. Among these patients, 8 patients were later diagnosed with AD in 19.0 ± 6.6 months after SPECT scan, and 30 patients were dementia-free for >3 years. Limited data has been published elsewhere^45^.

To demonstrate the feasibility of the proposed methods in a real clinical setting, a retrospective chart review study on a total of 113 patients was conducted. Subjects were patients of the Crescentwood Memory Clinic in Winnipeg, Manitoba, who were referred to the PET Centre at Health Science Centre (HSC) in Winnipeg, Manitoba, Canada, for scanning between 2010 and 2012 and subsequently diagnosed by a memory disorder specialist (PS) (Supplementary Fig. S2). HSC subjects were comprised of those who received a final diagnosis of MCI (n = 22), AD (n = 37), FTD (n = 4), DLB/PDD (n = 19) or VaD (n = 5) after having received a PET scan. Patients who were not followed for more than 6 months (n = 6) and who received other diagnoses (n = 21) were excluded. HSC cohort subject demographics are outlined in Table 5.

In order to test the performance of the proposed methods in other non-memory-oriented neurodegenerative disorders, data from a total of 97 subjects (18 NL, 17 PDND, 26 PD-MCI, 18 PDD, and 18 DLB) from a movement disorder clinic at Asan Medical Center in Seoul, South Korea were included (Table 6). All patients were scanned with FDG-PET, and their diagnoses were already well characterized at the time of scan. Limited data has been published elsewhere^26,39^.

All clinical and brain imaging data retrieval was completed in compliance with the Personal Health Information Act (PHIA), information regarding which can be found at http://www.gov.mb.ca/health/phia/. Ethics approval for this study was granted by the Biomedical Research Ethics Committee at the University of Manitoba, as well as institutional review boards at the Dong-A University Hospital and Asan Medical Center. Informed consent was obtained for all subjects who were enrolled in the study, either from the patient or from an individual designated as having power of attorney over the patient and able to make decisions regarding the patient’s health. All methods were performed in accordance with the relevant guidelines and regulations.

### Image Acquisition

ADNI FDG-PET images were retrieved from the ADNI Laboratory of Neuroimaging (LONI) database in a format under which images had already been preprocessed through coregistration, averaging, and standardization. The detailed procedure can be found at http://adni.loni.usc.edu/methods/pet-analysis/pre-processing/.

DongA University’s SPECT images were acquired as described previously^45^. Patients were injected with 925 MBq of Tc-99m-HMPAO at resting state. Brain SPECT images were obtained from patients using a dual-head gamma camera (Multi-SPECT II, ICON, Siemens, USA) equipped with fan-beamcollimator.

HSC’s FDG-PET images were acquired using Siemens Biograph 16 HiRez PET/CT (Siemens Medical Solutions, Knoxville, TN) scanner. Patients were fasted for at least 6 hours before scanning. Patients were injected i.v. with 185 MBq of FDG and a 15-minute static image was acquired starting 40 minutes post-injection. A head CT scan was acquired for attenuation correction purposes.

Asan Medical Center’s FDG-PET images were acquired as described previously^26,39^. All subjects fasted for at least 6 h before scanning. A 5-min transmission scan using a ^68^Ge rotating pin source and a 15-min emission scan were acquired on the ECAT HR + scanner (Siemens Medical Systems, Hoffman Estate, IL, USA) at the Asan Medical Center, 40 min after intravenous injection of 370 MBq of FDG.

### Image Preprocessing

All FDG-PET and perfusion SPECT image preprocessing was carried out using the standard procedure implemented in the statistical parametric mapping 12 (SPM) software (www.fil.ion.ucl.ac.uk/spm/) without the structural MRI (“old spatial normalization”). Images were normalized by warping to the MNI (Montreal Neurological Institute) standard space using a PET template available in SPM and then subsequently smoothed using an 8 mm Gaussian filter.

### GLM-based classifier

Following image pre-processing, all images were proportionally scaled to the mean of all voxels within the whole-brain mask that is generated by relative thresholding at 25% of maximum as previously described^13^, which resulted in 189,629 voxels. Linear regression (y = β × x + c; where y is the observed image, β is the slope coefficient, x is the dummy variables for the group, and c is the constant) was performed for each voxel with the dummy variables that classifies the subject groups (AD vs. NL). The β map was saved (Fig. 1A), which was used to calculate the subject scores by dot-product with the proportionally scaled brain images. Thus, the subject score represents how similar the brain image is in terms of visual appearance to the β map.

### SSM/PCA-based classifier

SSM/PCA was performed as described elsewhere^13^ using a customized MATLAB script. FDG-PET images of 111 NL and 94 AD subjects were log-transformed and double-centered. Singular value decomposition was performed to identify the top 10 PCs. The subject scores were calculated by dot-product between the scaled subprofile of each FDG-PET and PC. The PC that best discriminated the two groups was selected as the AD-related metabolic pattern for SSM/PCA1. For SSM/PCA2, stepwise regression was performed to select relevant PCs, then those PCs were linearly added with weights that were determined by the regression.

### SVM-based classifier

The same proportionally scaled images were used as in GLM. SVM analysis was done using the fitcsvm function with linear kernelling implemented in the MATLAB Statistical Toolbox – Machine Learning. Assuming a 5% false classification in the training set (i.e., patients with other types of dementia who were misdiagnosed of AD; normal subjects who may develop AD beyond the 3-year follow-up period), the outlier fraction was set at 5%. Other parameters remained at default for both ISDA (no assumption in the initial estimates, alpha; misclassification cost = [0 1; 1 0]; tolerance for gradient difference = 0; Feasibility gap tolerance = 0; maximal number of numerical optimization iterations = 1,000,000; kernel offset parameter = 0.1; kernel scale = 1) and SMO (no assumption in the initial estimates, alpha; misclassification cost = [0 1; 1 0]; tolerance for gradient difference = 0.001; Feasibility gap tolerance = 0; maximal number of numerical optimization iterations = 1,000,000; kernel offset parameter = 0; kernel scale = 1).

### Performance Evaluation and Statistical Analysis

The k-fold cross-validation (k = 10) was performed for all five methods (GLM, SSM/PCA1, SSM/PCA2, SVM-ISDA and SVM-SMO). For GLM and SSM/PCA, a receiver-operating curve (ROC) was examined to determine the optimal subject score threshold to produce the highest sensitivity and specificity in group discrimination. For SVM, the sign of the subject score was used to label each brain image as AD or non-AD. The performance characteristics of each classifier in group differentiation were evaluated by examining sensitivity × specificity.

The Student’s t-test was used to examine the group differences (AD vs. NL; stable MCI vs. prodromal MCI; across different neurodegenerative disorders) in age, MMSE, follow-up period, CSF markers (aβ, tau and phosphorylated tau), subject scores determined by GLM, SSM/PCA1 and SSM/PCA2. The subject scores (GLM and SSM/PCA) were compared by Student’s t-test between APOE ɛ4 carriers vs. non-carriers and male vs. female. These variables are correlated with Pearson’s correlation within each group. The differences is sex, APOE genotype and AD-designation (i.e., labels determined by SVM-ISDA and SVM-SMO) between groups are evaluated by chi-square test. All statistical analysis was performed using MATLAB R2014a and IBM SPSS Statistics version 24.

## Electronic supplementary material

Supplementary Information

## Data Availability

The five models (Fig. 1) produced in the current study can be accessed via http://www.kolabneuro.com/. The raw data are not publicly available with the exception of ADNI data as they contain patient medical data which can only be accessed under the Personal Health Information Act (PHIA), information regarding which can be found at http://www.gov.mb.ca/health/phia/.

## References

[1] McConathy, J. & Sheline, Y. I. Imaging Biomarkers Associated With Cognitive Decline: A Review. *Biol Psychiatry*, 10.1016/j.biopsych.2014.08.024 (2014). 10.1016/j.biopsych.2014.08.024 PMC4362908 25442005

[2] Klunk WE (2004). Imaging brain amyloid in Alzheimer’s disease with Pittsburgh Compound-B. Ann Neurol.

[3] Ma Y (2014). Predictive accuracy of amyloid imaging for progression from mild cognitive impairment to Alzheimer disease with different lengths of follow-up: a systematic review. Medicine.

[4] Pontecorvo MJ, Mintun MA (2011). PET amyloid imaging as a tool for early diagnosis and identifying patients at risk for progression to Alzheimer’s disease. Alzheimer’s research & therapy.

[5] Jagust W (2014). Time for tau. Brain.

[6] Partovi S (2017). Diagnostic performance of an automated analysis software for the diagnosis of Alzheimer’s dementia with (18)F FDG PET. Am J Nucl Med Mol Imaging.

[7] Shaffer JL (2013). Predicting cognitive decline in subjects at risk for Alzheimer disease by using combined cerebrospinal fluid, MR imaging, and PET biomarkers. Radiology.

[8] Presotto L (2017). Validation of (18)F-FDG-PET Single-Subject Optimized SPM Procedure with Different PET Scanners. Neuroinformatics.

[9] Chen X (2016). Potential Clinical Value of Multiparametric PET in the Prediction of Alzheimer’s Disease Progression. PLoS One.

[10] Yamane T (2014). Visual-statistical interpretation of (18)F-FDG-PET images for characteristic Alzheimer patterns in a multicenter study: inter-rater concordance and relationship to automated quantitative evaluation. AJNR. American journal of neuroradiology.

[11] Dukart J (2013). Meta-analysis based SVM classification enables accurate detection of Alzheimer’s disease across different clinical centers using FDG-PET and MRI. Psychiatry Res.

[12] Zhang D, Shen D, Alzheimer’s Disease Neuroimaging, I. (2012). Predicting future clinical changes of MCI patients using longitudinal and multimodal biomarkers. PLoS One.

[13] Spetsieris, P. *et al*. Identification of Disease-related Spatial Covariance Patterns using Neuroimaging Data. *J Vis Exp*, 10.3791/50319 (2013). 10.3791/50319 PMC3728991 23851955

[14] Tang CC (2010). Differential diagnosis of parkinsonism: a metabolic imaging study using pattern analysis. Lancet Neurol.

[15] Holtbernd F (2014). Abnormal metabolic network activity in REM sleep behavior disorder. Neurology.

[16] Niethammer M (2014). A disease-specific metabolic brain network associated with corticobasal degeneration. Brain.

[17] Cristianini, N. & Shawe-Taylor, J. xiii, 189 p. (Cambridge University Press, Cambridge; New York, 2000).

[18] Kecman, V., Huang, T. & Vogt, M. In *Support vector machines: theory and applications* (ed Lipo Wang) 255–274 (Springer, 2005).

[19] Fan RE, Chen PH, Lin CJ (2005). Working set selection using second order information for training support vector machines. J Mach Learn Res.

[20] Mosconi L (2005). Brain glucose metabolism in the early and specific diagnosis of Alzheimer’s disease - FDG-PET studies in MCI and AD. European Journal of Nuclear Medicine and Molecular Imaging.

[21] Mattis PJ (2016). Distinct brain networks underlie cognitive dysfunction in Parkinson and Alzheimer diseases. Neurology.

[22] Ko JH, Spetsieris P, Ma Y, Dhawan V, Eidelberg D (2014). Quantifying significance of topographical similarities of disease-related brain metabolic patterns. PLoS One.

[23] Peng S (2014). Characterization of disease-related covariance topographies with SSMPCA toolbox: Effects of spatial normalization and PET scanners. Hum Brain Mapp.

[24] Cabral C, Morgado PM, Campos Costa D, Silveira M (2015). & Alzheimers Disease Neuroimaging, I. Predicting conversion from MCI to AD with FDG-PET brain images at different prodromal stages. Comput Biol Med.

[25] Collij LE (2016). Application of Machine Learning to Arterial Spin Labeling in Mild Cognitive Impairment and Alzheimer Disease. Radiology.

[26] Ko JH, Lee CS, Eidelberg D (2017). Metabolic network expression in parkinsonism: Clinical and dopaminergic correlations. J Cereb Blood Flow Metab.

[27] Eidelberg D (2009). Metabolic brain networks in neurodegenerative disorders: a functional imaging approach. Trends Neurosci.

[28] Burns CM (2013). Higher serum glucose levels are associated with cerebral hypometabolism in Alzheimer regions. Neurology.

[29] Ishii K (2014). PET Approaches for Diagnosis of Dementia. American Journal of Neuroradiology.

[30] Kono AK (2007). Fully automatic differential diagnosis system for dementia with Lewy bodies and Alzheimer’s disease using FDG-PET and 3D-SSP. European Journal of Nuclear Medicine and Molecular Imaging.

[31] Chiba Y (2016). Early differential diagnosis between Alzheimer’s disease and dementia with Lewy bodies: Comparison between F-18-FDG PET and I-123-IMP SPECT. Psychiatry Research-Neuroimaging.

[32] Johnson KA (2004). Combined dopamine transporter and FDG PET IN DLB, AD, and PD. Neurobiology of Aging.

[33] Brosch JR, Farlow MR, Risacher SL, Apostolova LG (2017). Tau Imaging in Alzheimer’s Disease Diagnosis and Clinical Trials. Neurotherapeutics.

[34] Rinne JO (2000). Cognitive impairment and the brain dopaminergic system in Parkinson disease: [18F]fluorodopa positron emission tomographic study. Arch Neurol.

[35] Brück, A. *et al*. Positron emission tomography shows that impaired frontal lobe functioning in Parkinson’s disease is related to dopaminergic hypofunction in the caudate nucleus. *Neurosci Lett* **311**, 81–84, doi:S0304-3940(01)02124-3 (2001). 10.1016/s0304-3940(01)02124-3 11567783

[36] Kehagia AA, Barker RA, Robbins TW (2013). Cognitive impairment in Parkinson’s disease: the dual syndrome hypothesis. Neurodegener Dis.

[37] Nobili F (2010). Cognitive-nigrostriatal relationships in de novo, drug-naive Parkinson’s disease patients: a [I-123]FP-CIT SPECT study. Mov Disord.

[38] Burack MA (2010). *In vivo* amyloid imaging in autopsy-confirmed Parkinson disease with dementia. Neurology.

[39] Ko JH (2017). Distinct brain metabolic patterns separately associated with cognition, motor function, and aging in Parkinson’s disease dementia. Neurobiol Aging.

[40] Spetsieris PG (2015). Metabolic resting-state brain networks in health and disease. Proceedings of the National Academy of Sciences of the United States of America.

[41] Moeller JR (1996). The Metabolic Topography of Normal Aging. Journal of Cerebral Blood Flow & Metabolism.

[42] Udeochu JC, Shea JM, Villeda SA (2016). Microglia communication: Parallels between aging and Alzheimer’s disease. Clin Exp Neuroimmunol.

[43] Bu G (2009). Apolipoprotein E and its receptors in Alzheimer’s disease: pathways, pathogenesis and therapy. Nat Rev Neurosci.

[44] Iturria-Medina Y (2016). Early role of vascular dysregulation on late-onset Alzheimer’s disease based on multifactorial data-driven analysis. Nature Communications.

[45] Park KW (2012). Regional cerebral blood flow differences in patients with mild cognitive impairment between those who did and did not develop Alzheimer’s disease. Psychiatry Res.

