# Supplementary material for: Machine learning identified an Alzheimer’s disease-related FDG-PET pattern which is also expressed in Lewy body dementia and Parkinson’s disease dementia
Source: Sci Rep. 2018 Sep 5;8:13236. doi: 10.1038/s41598-018-31653-6 (PMC6125295; doi:10.1038/s41598-018-31653-6)
Supplement: Supplementary file 1 — Supplementary Information [file 41598_2018_31653_MOESM1_ESM.pdf]

SUPPLEMENTARY INFORMATION

for

Machine learning identified an Alzheimer's disease-related FDG-PET pattern which is also expressed in Lewy body dementia and Parkinson's disease dementia

Audrey Katako[1,2], Paul Shelton[3], Andrew L. Goertzen[4], Daniel Levin[4], Bohdan Bybel[4], Maram Aljuaid[1,2], Hyun Jin Yoon[5], Do Young Kang[5], Seok Min Kim[6], Chong Sik Lee[6,7], Ji Hyun Ko[1,2*]

[1]Department of Human Anatomy and Cell Science, Max Rady College of Medicine, Rady Faculty of Health Sciences, University of Manitoba, Winnipeg, Manitoba, Canada

[2]Neuroscience Research Program, Kleysen Institute for Advanced Medicine, Health Sciences Centre, Winnipeg, Manitoba, Canada

[3]Section of Neurology, Max Rady College of Medicine, Rady Faculty of Health Sciences, University of Manitoba, Winnipeg, Manitoba, Canada

[4]Section of Nuclear Medicine, Department of Radiology, Max Rady College of Medicine, Rady Faculty of Health Sciences, University of Manitoba, Winnipeg, Manitoba, Canada

[5]Department of Nuclear Medicine, College of Medicine, Dong-A University, Busan, South Korea

[6]Institute of Parkinson's Clinical Research, Ulsan University College of Medicine, Seoul, South Korea

[7]Department of Neurology, Asan Medical Center, Seoul, South Korea.

Table S1. Performance of AD vs. Normal classification methods using ADNI data that is spatially normalized with structural MRI by 10-fold cross-validation

	GLM	SSM/PCA1	SSM/PCA2	SVM-ISDA	SVM-SMO
**AUC**	0.8818	0.8711	0.8976	0.9269	0.9323
**Sensitivity**	0.9	0.7419	0.7097	0.8495	0.8495
**Specificity**	0.8182	0.9189	0.9459	0.9099	0.9099

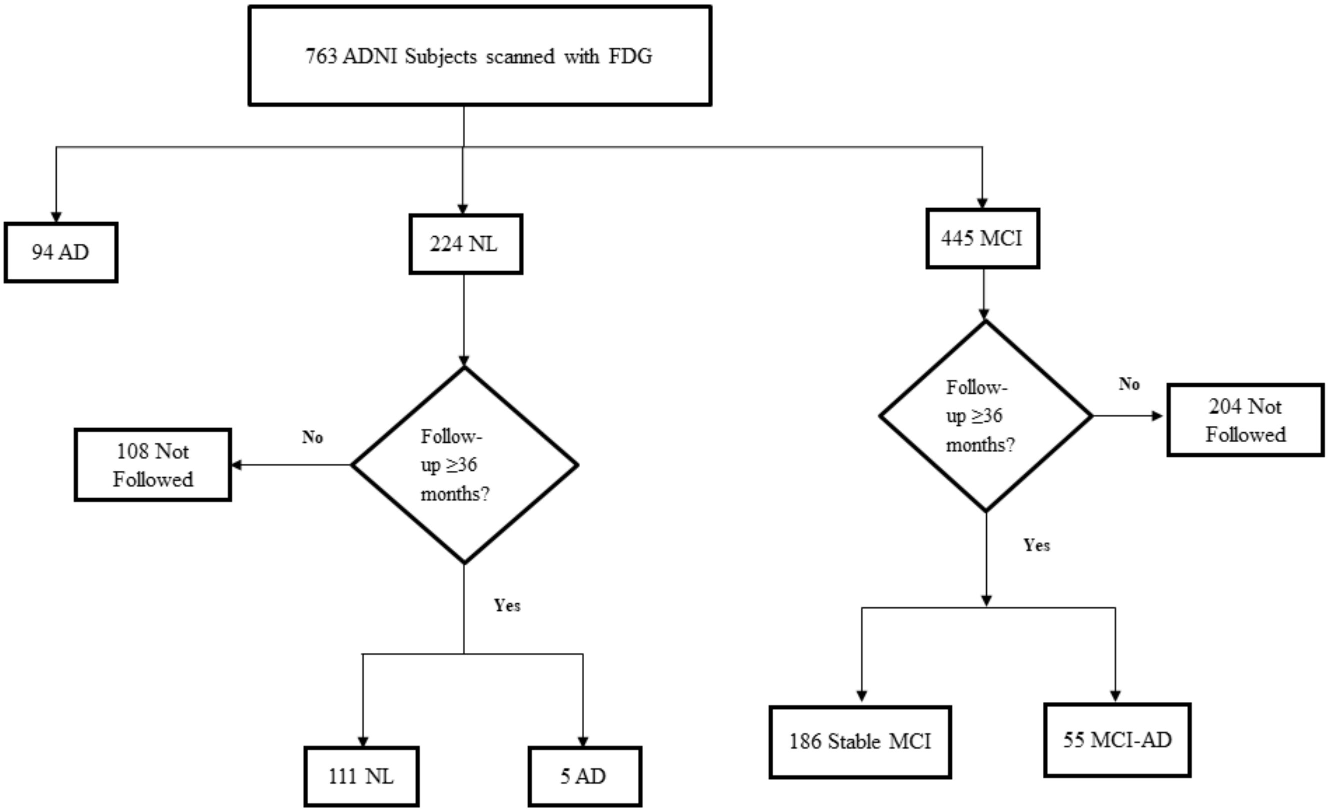

Figure S1: From the ADNI database, we identified three groups of individuals, i.e., patients with AD (AD; n=94), normal controls (NL; n=224), and subjects with mild cognitive impairment (MCI; n=445). Of the 445 MCI subjects selected for this study, 204 were not followed for a period of ≥36 months, and of the remaining 241, 186 remained stable MCI, with 55 subjects converting to AD (prodromal AD) within the 36-month follow-up period. Similarly, 224 NL subjects were studied, and of those 108 were not followed for ≥36 months. Of the remaining 116, 111 remained NL at the end of the follow-up period. Follow-up duration was neglected for AD subjects as dementia was already present at the time of screening.

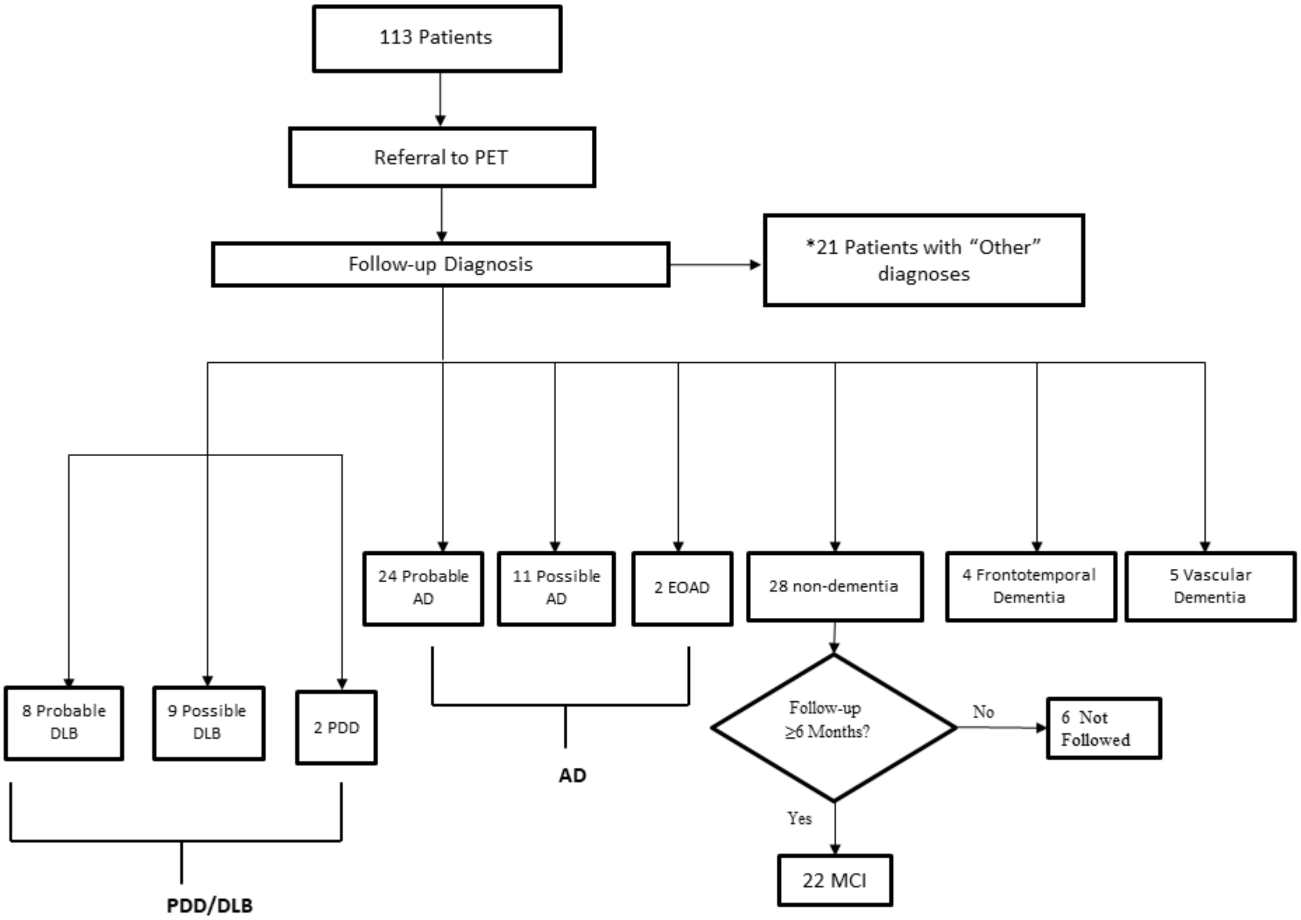

Figure S2: Schematic depicting the diagnostic progression of the study patients. 113 patients have been referred to our PET centre from Crescentwood memory clinic between 2010 and 2012. Final diagnosis was made based on clinical follow-up as well as FDG-PET and MRI (if applicable) report. 37 patients were subsequently diagnosed with Alzheimer's disease (AD) subjects. Of these, 24 had probable AD, 11 had possible AD, 2 had early-onset AD (EOAD; AD in patients younger than age 65). We also identified 17 patients subsequently diagnosed with Dementia with Lewy Bodies (DLB, 8 probable and 9 possible), 2 with Parkinson's disease Dementia (PDD), 4 with frontotemporal dementia (FTD), and 5 with vascular dementia (VaD). 21 patients received other diagnosis which include hydrocephalus, corticobasal syndrome, aphasia, comorbid AD/VaD, and closed head injury. 28 patients were not diagnosed with any specific condition other than mild cognitive impairment (MCI). Of these, 22 were followed for >6 months.
